# Step-by-step guide for electrochemical generation of highly oxidizing reactive species on BDD for beginners

**DOI:** 10.3389/fchem.2023.1298630

**Published:** 2024-01-04

**Authors:** G. Xavier Castillo-Cabrera, Caroline I. Pliego-Cerdán, Erika Méndez, Patricio J. Espinoza-Montero

**Affiliations:** ^1^ Escuela de Ciencias Químicas, Pontificia Universidad Católica del Ecuador, Quito, Ecuador; ^2^ Facultad de Ciencias Químicas, Benemérita Universidad Autónoma de Puebla, Puebla, Mexico

**Keywords:** electrochemical oxidation, highly oxidizing reactive species, sampled current voltammetry, boron-doped diamond, Tafel plot, amoxicillin degradation

## Abstract

Selecting the ideal anodic potential conditions and corresponding limiting current density to generate reactive oxygen species, especially the hydroxyl radical (^•^OH), becomes a major challenge when venturing into advanced electrochemical oxidation processes. In this work, a step-by-step guide for the electrochemical generation of ^•^OH on boron-doped diamond (BDD) for beginners is shown, in which the following steps are discussed: i) BDD activation (assuming it is new), ii) the electrochemical response of BDD (in electrolyte and ferri/ferro-cyanide), iii) Tafel plots using sampled current voltammetry to evaluate the overpotential region where ^•^OH is mainly generated, iv) a study of radical entrapment in the overpotential region where ^•^OH generation is predominant according to the Tafel plots, and v) finally, the previously found ideal conditions are applied in the electrochemical degradation of amoxicillin, and the instantaneous current efficiency and relative cost of the process are reported.

## 1 Introduction

When delving into the wide world of advanced oxidation processes (AOPs), we found an overwhelming amount of information that can be frustrating for a beginner. All these technologies mainly seek the generation of the hydroxyl radical (^•^OH) and other highly oxidizing reactive species (HORS) to carry out highly efficient oxidation processes. Advanced electrochemical oxidation (AEO) is one of the most studied AOPs for the oxidation/degradation of organic matter in aqueous media (Martínez-Huitle et al., 2015; Bessegato et al., 2021; Hand and Cusick, 2021; Pierpaoli et al., 2021; Espinoza-Montero et al., 2023). AEO consists of applying a polarization potential to a conductive substrate (metal or semiconductor) to produce HORS, mainly ^•^OH if possible, on the electrode–solution interface (Ganiyu et al., 2021; Lee et al., 2022; Xu et al., 2023b). One of the most appreciated electrodes for AEO is the boron-doped diamond (BDD) electrode. BDD is a p-type semiconductor due to the chemical nature of boron, as they are impurity/dopant donors, so in thermal equilibrium, there is an imbalance of charge carriers in the valence and conduction bands, with the positive holes being the majority carriers (Yokoya et al., 2005; Yang et al., 2019). As an electrode, BDD is a very versatile material as it has unique electronic properties. It is a non-active electrode; *i.e.*, its chemical structure is not compromised by the redox processes to which it may be subjected, although its reticular terminations are susceptible to slight changes during oxidation and reduction (Berenguer et al., 2019). As it has a very wide potential, this electrode has been used for multiple applications in electroanalytical chemistry (Wei et al., 2011b; Joshi et al., 2022), electrosynthesis (Ivandini and Einaga, 2017; Lips and Waldvogel, 2019), and AEO for water treatment (Cisneros-León et al., 2023).

For water treatment, electrochemical oxidation can take advantage of the complexity of mixtures of organic and inorganic species that can be found in typical contaminated water since, depending on the composition of the inorganic electrolyte in the system to be treated, the so-called HORS can be generated (Wen et al., 2017; Xu et al., 2021; Ahmed et al., 2022; Parvulescu et al., 2022; dos Santos et al., 2023; Mousset, 2023). Most HORS are “free” radicals and detrimental to most biological systems; the opposite is true for advanced oxidation processes, where they contribute to the oxidation of organic and inorganic matter (Murphy et al., 2022; Cisneros-León et al., 2023). HORS covers a wide spectrum of inorganic radicals, ions, and neutral molecules in aqueous media whose redox potentials are varied (Ross and Neta, 1982; Armstrong et al., 2015). Directly or indirectly, electrochemical oxidation can generate reactive oxygen species (ROS) such as the superoxide radical (O_2_^•–^), singlet oxygen (^1^O_2_), hydrogen peroxide (H_2_O_2_), and the strongest oxidant, the hydroxyl radical (^•^OH) (Xie et al., 2022). On the other hand, in the presence of nitrates and nitrites, through the reaction of the electrogenerated ^•^OH, reactive nitrogen species (RNS) such as mono-, di-, and trioxide nitrogen radicals (NO^•^, NO_2_^•^, and NO_3_^•^) can be obtained (Wu et al., 2020; Rayaroth et al., 2022). Likewise, in the presence of sulfates, sulfites, and carbonates, sulfate (SO_4_^•–^) and sulfite (SO_3_^•–^) radicals, the persulfate anion (S_2_O_8_^2–^), and the carbonate radical (CO_3_^•^) can be generated *in situ*, and in the presence of chlorides or chlorites, reactive chlorine species (RCS) are very common, such as the chlorine radical (Cl^•^) and the oxychloride radical (ClO^•^) (Ganiyu and Martínez-Huitle, 2019; Ganiyu et al., 2021; Zhou and Xiao, 2022). In addition, reactive phosphate species (RPS) are generated indirectly by reacting ^•^OH with phosphates, producing phosphate radicals in three acidic forms, the H_2_PO_4_^•^ radical being the most powerful oxidizing species of this type (Weiss et al., 2008). All these species can contribute greatly to the oxidation of organic and inorganic pollutants, achieving a predominant synergistic effect, or they can have the opposite effect since they all compete in coupled reactions in electrocatalysis.

This work intended to provide a step-by-step guide aimed primarily at beginners in the investigation of electrode properties in common electrolytic systems, with emphasis on the HORS that can be generated in the medium and that compete in catalysis. First, an approach from the existing theory in this field is addressed, and then, a real example is given using the BDD electrode in a known electrolytic environment.

## 2 Step-by-step electrooxidation on BDD

To experimentally determine the optimal operating conditions at AEO, some fundamental electrochemical tests in known redox environments must be considered. In the following, we will carefully describe the suggested minimum steps that will allow us to develop our understanding of the optimal conditions for electrolysis at larger scales.

### 2.1 Electrode activation and cleaning (if required)

BDD is a “non-active” electrode when acting as an anode, and the HORS that are generated when a high oxidation overpotential is applied do not drastically change their chemical nature; this property makes it a unique material and is the basis for a large number of applications including in the area of environmental remediation (Sánchez-Montes et al., 2020; Carrera-Cevallos et al., 2021; Cisneros-León et al., 2023; Long et al., 2023). Therefore, studying the surface terminations of the crystal lattice of this material becomes key and is addressed in this section.

**FIGURE 1:**
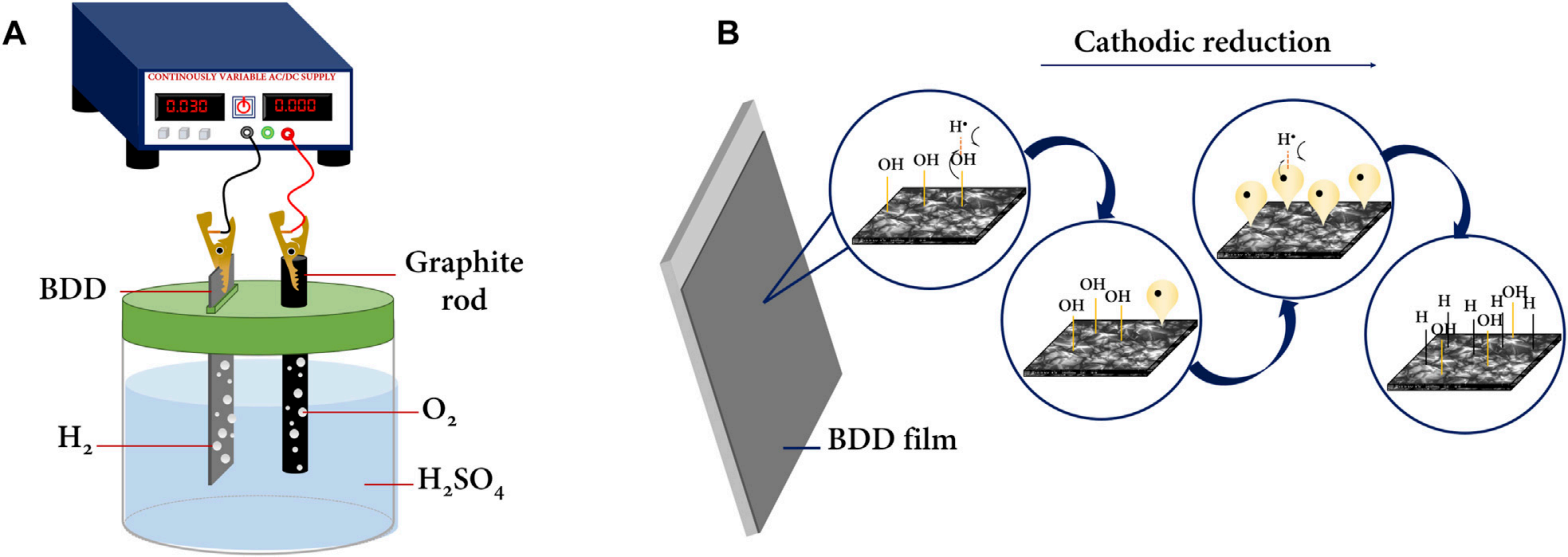
**(A)** Two-electrode cell for electrolysis in an acidic medium performed in lab. **(B)** BDD surface transition by cathodic reduction, adapted from the work of Einaga (2022).

Diamond is a purely insulating material since its crystal lattice is composed of hybridized C-sp^3^. However, by introducing impurities of boron or nitrogen atoms during its synthesis using controlled methods at high temperatures and pressure, such as chemical vapor deposition (CVD) (Macpherson, 2015; Sun et al., 2021), a conducting, semiconducting, or superconducting material is obtained as a result of two factors; the first is that by introducing boron (or nitrogen) atoms, carriers that previously formed bonds are “released” and move freely through the lattice and participate in conduction in the BDD (Yokoya et al., 2005; Yang et al., 2019). On the other hand, when impurities are introduced to the diamond, it changes the hybridization of certain carbon atoms (from C-sp^3^ to Csp^2^), which extends to the surface terminations of the crystal lattice. The sp^2^ (C-sp^2^) hybridization (graphitic carbon) is the one that contributes the most to the conductive properties of BDD. Therefore, the surface termination of the BDD is a mixture of C-sp^3^ and C-sp^2^. When the surface termination is predominantly C-sp^3^, the BDD is said to have an H-termination, while when the surface termination is C-sp^2^, it is said that the surface termination is predominantly oxygen or oxygenated organic functional groups (Medeiros De Araújo et al., 2014; Garcia-Segura et al., 2015). Surface modifications of BDD from C-sp^2^ to C-sp^3^ or *vice versa* are possible through different controlled experimental methods. BDD electrode manufacturers often prefer methods that are as effective as their cost, *e.g.*, oxygen plasma for oxygenated surfaces and hydrogen plasma for hydrogenated surfaces, but these are not practical for use in most laboratories (Kasahara et al., 2017; Einaga, 2022). On the other hand, surface modification of BDD electrochemically is simpler and cheaper; *e.g.*, anodic polarization generates an oxygen surface termination, and cathodic polarization generates a hydrogen surface termination, for which a power source and a two-electrode cell are required (Figure 1A). This simple assay is commonly performed in strong acid media (H_2_SO_4_, HClO_4_, and HNO_3_) at moderate concentrations. The transitions from H to O or *vice versa*, in addition to the intrinsic equilibrium of adsorption–desorption of the gases produced on the surface—O_2_ at the anode and H_2_ at the cathode—are achieved as long as the redox potentials of the organic forms present in the lattice are reached; *i.e.,* care must be taken to control the DC density applied to the cell as well as the time for which the electrode is exposed to oxidation or reduction.

Einaga (2022) proposed a mechanism for the transition from oxygenated to hydrogenated BDD terminations. In essence, there is a homolytic contribution of hydrogen and the –O–H group from the BDD surface, yielding a good leaving group (water). In this way, the carbon formerly bonded to the R–CH_2_–O–H group leaves a hybrid orbital with an unpaired electron ripe to overlap with another atomic hydrogen and obtain surface groups with hydrogen-bonded carbons (R–CH_2_ **–**H) (Figure 1B). In addition, electrolysis in a strong acidic medium serves to clean impurities from BDDs prior to use, if required. It is very common to adsorb carbon impurities generated during the synthesis of the BDD or by the adsorption of contaminants from the environment or from contact with other surfaces. Therefore, it is common that during the manufacture of BDDs, especially bipolar (double-sided modified) BDDs, a thin layer of hybrid material with major impurities remains, and electrochemical cleaning is useful to remove this layer, as recommended by the manufacturers themselves. Cleaning, which should be understood interchangeably as surface activation of the BDD, is a soft electrochemical method that does not compromise the crystalline integrity of the BDD, much less of the substrate on which the doped diamond layer has been deposited, which is commonly Nb or n-type Si.

### 2.2 Electrode characterization

**FIGURE 2:**
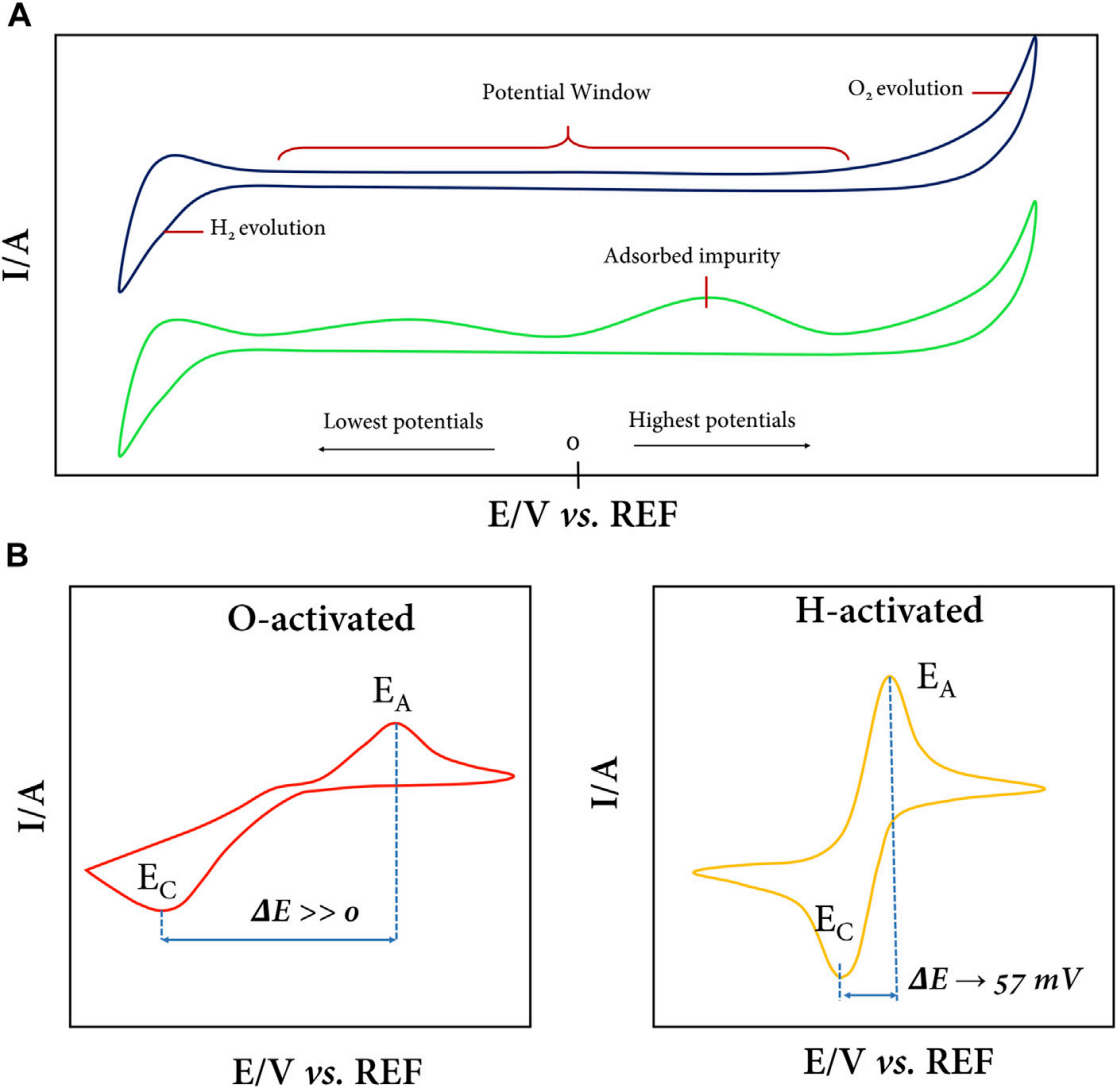
**(A)** Voltammetric profiles of the full potential window of the BDD in the presence of an electrolyte. Without the presence of contaminants or adsorbed impurities, only the oxygen and hydrogen evolution peaks are observed (top), while on a soiled surface, peaks are present in the capacitive zone (bottom). **(B)** Typical BDD CVs in the ferri/ferro-cyanide redox couple for an oxygenated (left) and hydrogenated (right) surface.

By cyclic voltammetry (CV), the cleanliness and surface termination of the BDD can be evaluated, as shown in Figure 2A. The BDD has an impurity-free surface (Figure 2A top) when no peak appears in the potential range of the capacitive region, and only the extreme peaks of oxidation evolution (more positive potentials) and hydrogen evolution (more negative potentials) should be detected. However, additional signals characteristic of surface carbon impurities often appear when the electrode is not properly cleaned/activated (Figure 2A bottom). These CV profiles are typically recorded in the presence of the electrolyte only, commonly Na_2_SO_4_, H_2_SO_4_, and phosphate buffer solutions, in dilute concentration (0.1 M). Although it is also possible to use strong bases (NaOH or KOH) as the supporting electrolyte to generate these voltammograms, caution must be exercised when using a suitable reference electrode (Zoski, 2007).

CV, in addition to being useful for calculating the electrode area by applying the Randles–Sevcik equation, is also useful for evaluating the predominant surface termination of H or O on BDD after electrochemical treatment. For this purpose, one can use the [Fe(CN)_6_]^3–^/[Fe(CN)_6_]^4–^ redox couple (or others) in dilute concentration and in a suitable electrolyte medium, typically KCl in a 1:100 ratio, although it is common to use more concentrated electrolyte solutions to avoid potential losses due to Ohmic drop (Suffredini et al., 2004; Actis et al., 2008; Zanin et al., 2014; Cheah and Chernev, 2021; Bard et al., 2022; Zheng, 2023). Figure 2B shows the typical CVs of BDD electrodes in the ferro/ferri-cyanide redox couple. At an electrode with oxygen surface terminations (Figure 2B left), the [Fe(CN)_6_]^3–^/[Fe(CN)_6_]^4–^ redox process becomes very slow, and the separation of anodic potential (E_a_) and cathodic potential (E_c_) peaks is enormous; *i.e.*, ΔE_p_ (E_c_- E_a_) takes large values. On the contrary, in Figure 2B (right), a typical CV of a BDD electrode with H-terminations predominant on the surface is observed, where the redox couple response is fast, chemically and electrochemically reversible, ΔE_p_ is close to 57 mV, and the width at half on the forward scan of the peak is close to 59 mV (Elgrishi et al., 2018). In practice, it is crucial to define which surface is of interest depending on the intended application of the BDD electrode (Oliveira et al., 2010; Kondo, 2022). For example, if a semiconductor-photocatalyst-modified electrode is required, it is ideal to have an oxidized surface as the physical and chemical interactions are stronger for oxygen with the semiconductor metal. On the other hand, if bare electrode electrocatalysis is what is sought, a predominantly hydrogenated surface is ideal since charge transfer at the solid–liquid interface is favored.

### 2.3 Selecting parameters

Once BDD is electrochemically characterized to “micro-scale,” the next step is the search for the “ideal” parameters to carry out electrochemical oxidation with the greatest efficiency, driven mainly by HORS, which will be discussed in depth later. AEO is a complex process involving the balance of several variables that must be carefully controlled in practice (Ganiyu et al., 2021; Bany Abdelnabi et al., 2022; Lee et al., 2022; Xu et al., 2023a; Zheng, 2023). In this section, we will focus mainly on the potential and/or current density to be applied to the system and how the electrochemical test should be carried out to find it. Our purpose is to systematically relate the Tafel analysis to the different electrolyte species that may be present and their consequent formation of HORS at the BDD electrode. For the construction of the Tafel curves, we will use voltammograms generated by sampled current voltammetry, a very effective and easy-to-apply technique.

#### 2.3.1 Tafel analysis

To monitor an electrochemical process quantitatively and from the point of view of kinetics, the Tafel analysis is often very useful for certain processes (Bard et al., 2022; Shih et al., 2022). The equation that quantitatively relates the potential to the current of a specific system is called the Tafel equation (Eq. 1), and it was first designed to study the hydrogen evolution reaction (HER) (Tafel, 1905; Petrii et al., 2007; Fang and Liu, 2014; Jung et al., 2022).

$$\eta =a-b\ln j\;or\;\eta =\frac{RT}{\alpha F}\ln{\;i}_{0}-\frac{RT}{\alpha F}\ln j,$$ (1)

where η is the overpotential, which is calculated from the algebraic subtraction of the potential applied ( $E_{app}$ ) to the working electrode and the open-circuit potential ( $E_{OCP}$ ), *i.e.,* the equilibrium potential that is specific for each electrolyte in solution. The Tafel plot is obtained by plotting ln⁡j vs. η , where *b* is the Tafel slope(s), which is of interest to us because of its kinetic significance (Fang and Liu, 2014).

To study the kinetics of redox processes in electrochemistry, the rotating disk electrode is employed since the speed of revolution is controlled and the diffusional and capacitive influences of the system are eliminated, thus allowing the kinetic parameters and charge transfer constants to be calculated with high reproducibility (Chen et al., 2020). However, not all laboratories have this sophisticated system at their disposal. Instead, sampled current voltammetry (SCV) is a useful technique that frees us from this experimental drawback, and the kinetics of reversible or irreversible reactions can be studied with the Tafel analysis without major problems (Soares et al., 2020; Rodríguez and Denuault, 2021).

**FIGURE 3:**
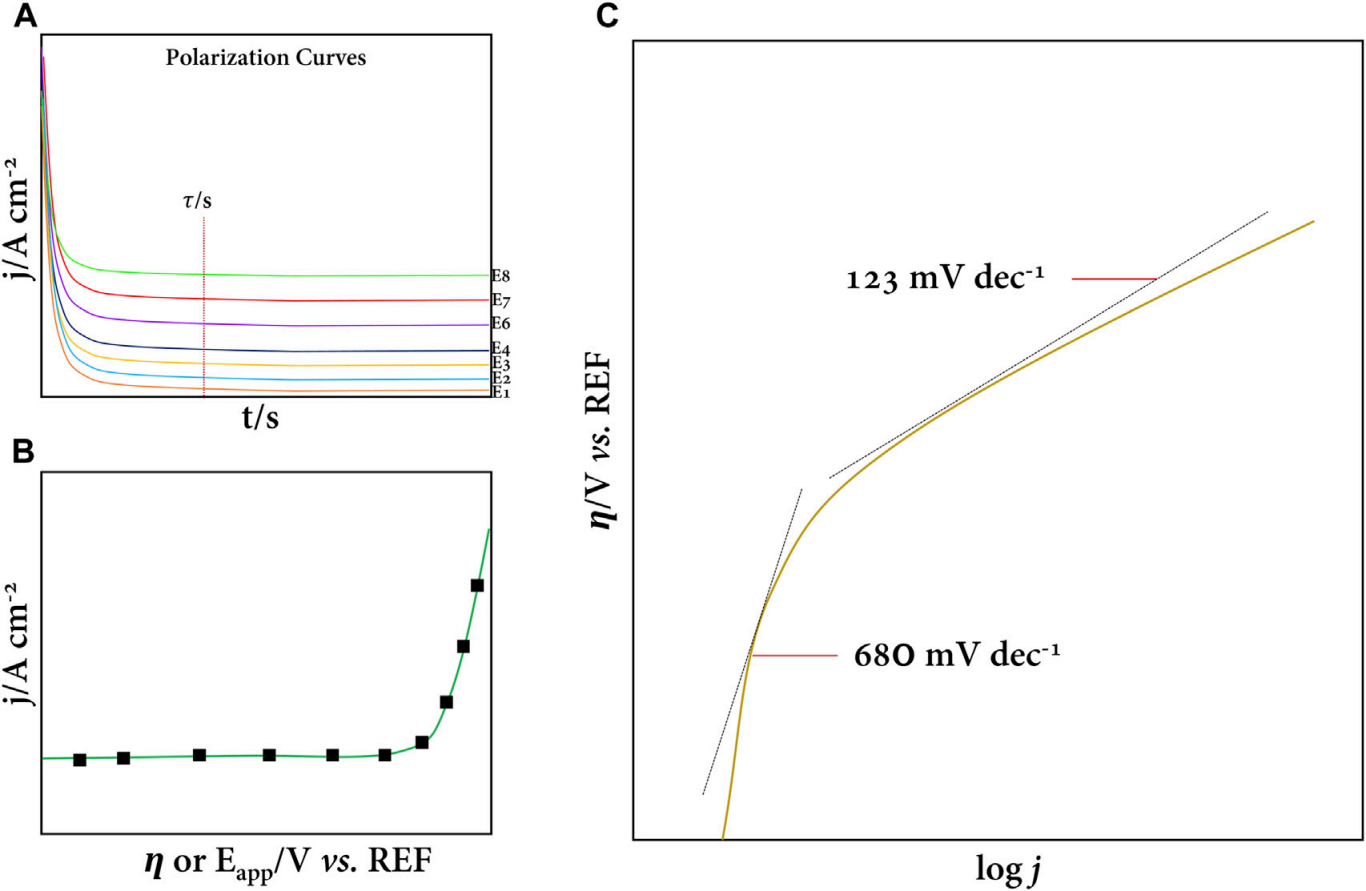
**(A)** Typical polarization curves at oxidation overpotentials in a given electrolyte. **(B)** j vs. η curve corresponding to OER. **(C)** Tafel plot for OER. **(C)** Adapted from the work of Kapałka et al. (2008).

An SCV assay consists of subjecting the working electrode to various polarization potentials—in a specific range and in the medium of interest—during a specific pulse time (chronoamperometry); in this sense, the so-called polarization curves are obtained (Figure 3A). The polarization curves are used to select the current density corresponding to each potential at a given time constant ( τ ), thus plotting the η vs. j or E_app_ vs. j curve (Figure 3B) and applying the Tafel analysis to the data obtained.

Kapałka *et al.* (2008) pioneered the study of the kinetics of the oxygen evolution reaction (OER) at a BDD electrode (Figure 3C). They showed that in the Tafel analysis, there were two well-defined slopes corresponding to independent, one-to-one electron transfer processes. For our interest and depending on the electrolyte, the Tafel analysis gives us a hint of the optimal potential to produce HORS before oxygen evolution is favored (thermodynamically). Therefore, this kinetic study will allow us to select a wide range of anodic potentials and evaluate the production of HORS, as discussed below.

#### 2.3.2 HORS at BDD

A further digression is necessary here to address the HORS generated on the BDD. Depending on the electrolyte in which the electrolysis is carried out, different reactive species capable of efficiently oxidizing organic molecules may coexist, some more than others. We cannot begin this discussion without first analyzing the ROS and, in essence, the hydroxyl radical (^•^OH). This radical is the first intermediate of the OER from water splitting (Reaction 1), and it is generated in a process favored by kinetics rather than thermodynamics; i.e., large overpotentials are required for its formation on the BDD surface (Siahrostami et al., 2017; Zhu, 2019; Xie et al., 2022).

$$\text{BDD}+H_{2}O+E_{\text{app}}\to \text{BDD}\left({\text{OH}}^{*}\right)+H^{+}+1e^{-}$$ (1)

**FIGURE 4:**
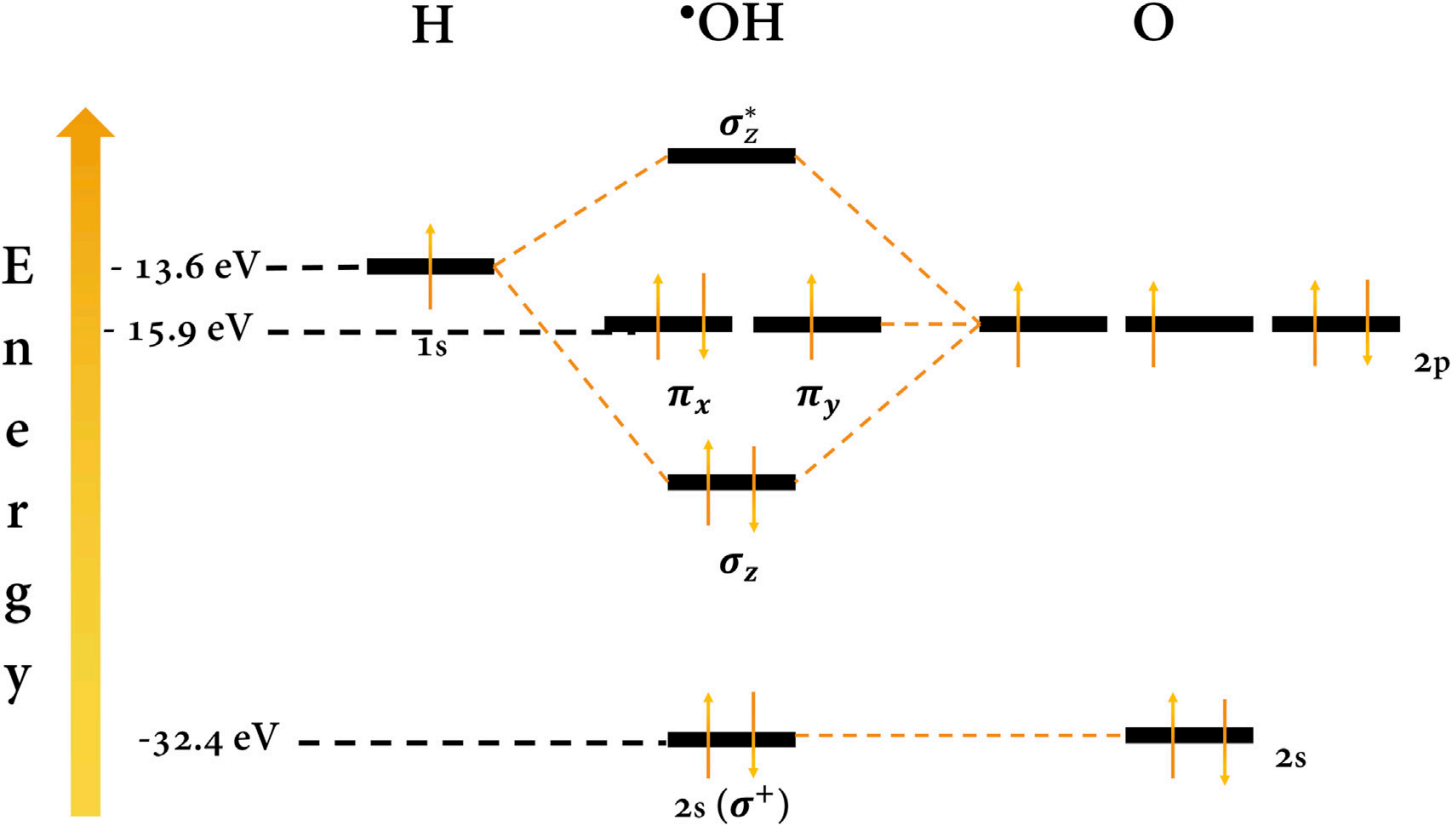
Molecular orbital diagram for the ^•^OH. Energy values were taken from the work of Mann et al. (2000).

BDD (OH*) refers to the ^•^OH adsorbed on the surface of the BDD, specifically on the active site, which plays a crucial role (Moreira et al., 2017; Vogt and Weckhuysen, 2022). ^•^OH is a highly reactive and unstable species whose lifetime is estimated to be a few nanoseconds (Moreira et al., 2017). The high reactivity of the ^•^OH is explained by its unpaired electron in an antibonding orbital (π*) of an oxygen atom located at high energy (Figure 4). This instability (in energy) means that its electron easily pairs with an organic species to form covalent bonds in the electrooxidation pathway (Imlay, 2003; Krumova and Cosa, 2016). Its oxidizing power (∼2.73 V vs. NHE), second only to fluorine, is due to its high tendency to capture electrons and stabilize the antibonding orbital. Therefore, it is capable of non-selectively oxidizing a wide variety of organic pollutants as long as the redox potential of the target molecules is aligned with the redox potential of the radical at the interface (Armstrong et al., 2015).

**FIGURE 5:**
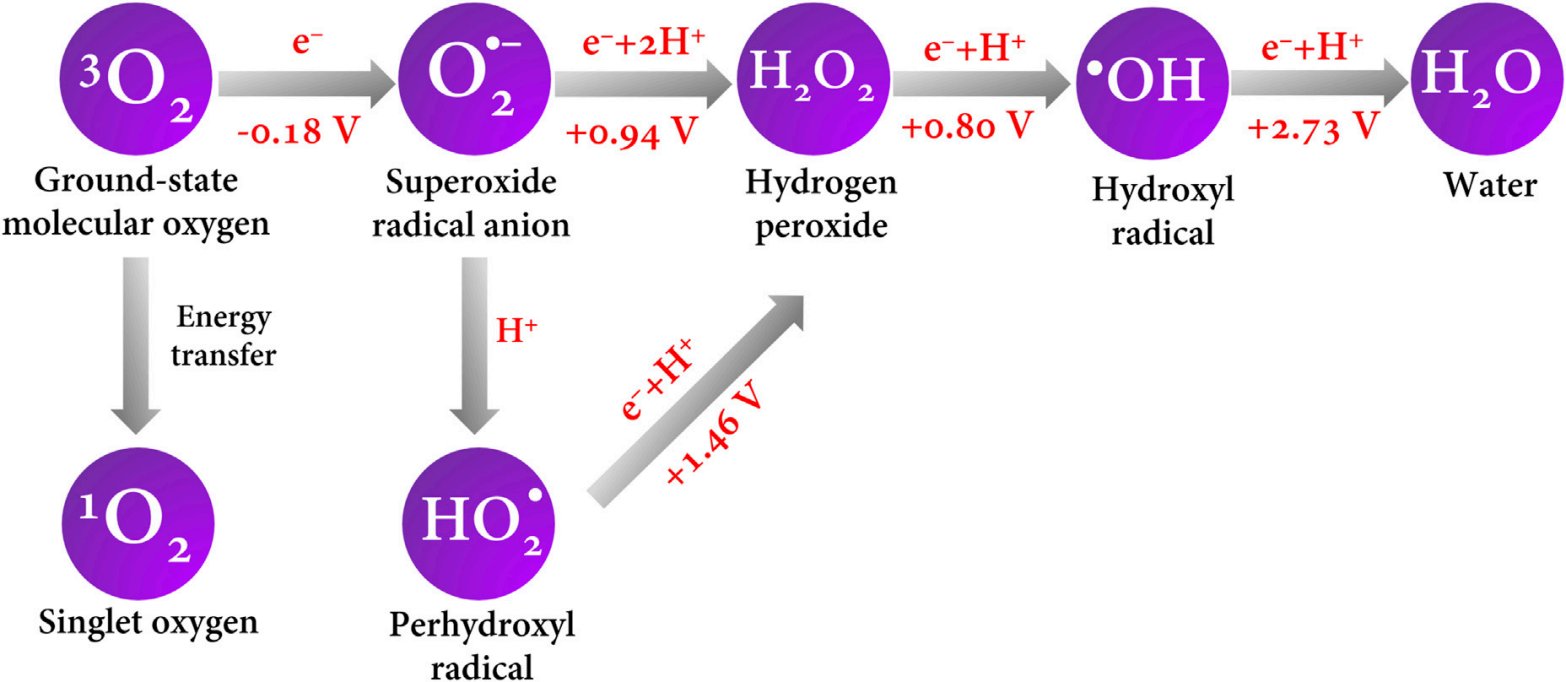
ROS formation by electron and energy transfer. Redox potential values are reported vs. NHE and were taken from the work of Armstrong et al. (2015).

On the other hand, molecular oxygen in its fundamental state is triplet (^3^O_2_) and is dissolved in the medium or generated by the electrolysis of water; it can trigger reduction reactions to generate other highly reactive species but with lower oxidizing power than the ^•^OH (Krumova and Cosa, 2016). When O_2_ is reduced by the conduction band electrons of the BDD, on transfer of a first electron, it produces the superoxide anion radical O_2_^•–^ (E^0^ = −0.18 V vs. NHE) (Armstrong et al., 2015; Hayyan et al., 2016). In brief, Figure 5 shows a schematic of the ROS generated by electron transfer and their redox potentials.

In short, the ^•^OH, due to its oxidizing power, is the species that is sought to be generated efficiently in AEO, and it will be seen from now on that this radical plays a crucial role in the generation of other highly reactive oxidizing species (Gligorovski et al., 2015). As for pH, it is also a parameter that greatly influences the generation of reactive species and electrocatalysis in general. We said that the ^•^OH production process is kinetically favorable, where pH influences the rate of charge transfer (Tao et al., 2019; Zhang et al., 2020). At low pH, the concentration of H_3_O^+^ increases, which favors the generation of ^•^OH. H_3_O^+^ can react with electrons from the electrode (depending on the electrode potential) and produce atomic hydrogen, which is highly reactive and reacts with water to produce ^•^OH (Katsounaros et al., 2014). Very low pH can also be detrimental to the electrode as it can create a passivation layer on the electrode surface that limits charge transfer; in BDD, this is not a drawback as its high stability in strong acid environments frees it from this issue (Xie et al., 2022). Other considerations must be taken with respect to pH since the stability of the target molecule to be degraded is also a tremendously influential factor, as well as the concentration of the electrolyte species in the solution (Fornaciari et al., 2022).

The utility of ions in a solution is imperative for the vast majority of electrochemical processes (Bard et al., 2022). In the process at stake here, the supporting electrolyte plays a dual role since it is not only responsible for mitigating the migration of the electroactive species and serving as an ionic conductor in the medium but the ions can also react instantaneously with the electrogenerated ^•^OH and produce other chemical species of high reactivity and, sometimes, high oxidizing power (Kim et al., 2018; Hand and Cusick, 2021). Many authors refer to this process as the “activation” of a certain species since in the absence of any perturbation, the ions are not a major threat to organic pollutants (Lin and Deng, 2021). In a medium containing sulfate ions, activation of the sulfate radical ( ${\text{SO}}_{4}^{•-}$ ) and other reactive sulfate species is very likely. This radical anion is mainly produced by the oxidation of ( ${\text{SO}}_{4}^{2-}$ ) in the presence of ^•^OH adsorbed on the BDD in a 1-electron transfer process (Reaction 2). Another species, the persulfate anion ( ${S_{2}O}_{8}^{2-}$ ), can be generated by direct oxidation on the surface of the BDD or by oxidation of sulfate by the ^•^OH in a 2-electron transfer process (Reaction 3) or by direct combination of two sulfate radicals (Reaction 4) (Oh et al., 2016; Lee et al., 2020; Ganiyu et al., 2021; Araújo et al., 2022).

$$\text{BDD}\left({\text{OH}}^{*}\right)+{\text{SO}}_{4}^{2-}\to \text{BDD}\left({\text{SO}}_{4}^{•-}\right)+{\text{OH}}^{-}$$ (2)

$$2\text{BDD}\left({\text{OH}}^{*}\right)+{2\text{SO}}_{4}^{2-}\to \text{BDD}\left({S_{2}O}_{8}^{2-}\right)+2H_{2}O$$ (3)

$$2\text{BDD}\left({\text{SO}}_{4}^{•-}\right)\to \text{BDD}\left({S_{2}O}_{8}^{2-}\right)$$ (4)

In the literature, the vast majority of works that seek to treat polluted water by electrochemical oxidation use sulfate-based electrolytes, typically sodium or potassium sulfate, or sulfuric acid. Why is this? This can be accounted for by the oxidizing power of this radical, which ranges from 2.5 to 3.1 V vs. NHE; these values compete with the oxidizing power of the ^•^OH (Ganiyu and Martínez-Huitle, 2019; Ganiyu et al., 2021; Araújo et al., 2022). When comparing these two species, both radicals have an affinity for capturing electrons in redox processes since they have unpaired antibonding molecular orbitals in oxygen, while the electronegativity of S is greater than that of O, and the polarization of the bond is more efficient (Lin and Deng, 2021). Therefore, if these two species can coexist in a moderate “balance,” the treatment of contaminated water can be very efficient. Understanding the oxidation power of these species from theory can be a simple issue, but, in practice, it is not so since variables such as pH, applied potential and current density, temperature, and contaminants must be controlled.

Reactive chlorine species (RCS) are produced in an electrolytic chloride environment. Mainly, the chlorine radical (Cl^•^) is the outstanding species of this group with an oxidation power of approximately 2.43 V vs. NHE (Armstrong et al., 2015). It is produced by indirect oxidation, *i.e.*, by the oxidation of Cl^−^at the electrode surface by the adsorbed ^•^OH (Reaction 5) (Mostafa et al., 2018). On the other hand, the hypochlorite radical (ClO^•^), also a highly reactive species of chlorine, is produced by the same oxidation reaction by the ^•^OH (Reaction 6) and has a relatively low oxidation power (1.39 V vs. NHE) (Armstrong et al., 2015). The ^•^OH can trigger reactions with chloride in the medium to produce hypochlorite, chlorite, chlorate, and perchlorate anions, but they are not highlighted here since their oxidizing power is overshadowed by their toxicity, and reactions producing these species can be found anywhere (Ganiyu et al., 2021; Choo et al., 2022; Barnum and Coates, 2023).

$$\text{BDD}\left({\text{OH}}^{*}\right)+{\text{Cl}}^{-}\to \text{BDD}\left({\text{Cl}}^{•}\right)+{\text{OH}}^{-}$$ (5)

$$\text{BDD}\left({\text{OH}}^{*}\right)+{\text{ClO}}^{-}\to \text{BDD}\left({\text{ClO}}^{•}\right)+{\text{OH}}^{-}$$ (6)

Similarly, reactive phosphate species (RPS) are produced by hydroxyl radical chain reactions (Reactions 7–9). The phosphate radical ( ${\text{PO}}_{4}^{•2-}$ ) can exist in its three acidic forms, with the radical ( $H_{2}{\text{PO}}_{4}^{•}$ ) having the highest oxidizing power, approximately 2.75 V vs. NHE (Armstrong et al., 2015). The other radical species are less reactive for both redox processes and acidic proton capture (Ross and Neta, 1982; Weiss et al., 2008).

$$\text{BDD}\left({\text{OH}}^{*}\right)+H_{3}PO_{4}⇄\text{BDD}\left(H_{2}{\text{PO}}_{4}^{•}\right)+H_{2}O$$ (7)

$$\text{BDD}\left({\text{OH}}^{*}\right)H_{2}{\text{PO}}_{4}^{-}⇄\text{ BDD}\left(H_{2}{\text{PO}}_{4}^{•}/\;H_{2}{\text{PO}}_{4}^{•-}\right)+{\text{OH}}^{-}/H_{2}O$$ (8)

$$\text{BDD}\left({\text{OH}}^{*}\right)+H{\text{PO}}_{4}^{2-}⇄\text{ BDD}\left({\text{HPO}}_{4}^{•-}/\;{\text{PO}}_{4}^{•2-}\right)+{\text{OH}}^{-}/H_{2}O$$ (9)

Finally, it is not difficult to imagine that reactive nitrogen and carbon species are produced by the indirect oxidation of the hydroxyl or sulfate radical with nitrates, nitrites, and carbonates to generate nitrogen di- and trioxide radicals ( ${\text{NO}}_{2}^{•},{\text{NO}}_{3}^{•}$ ), as well as the carbonate radical ( ${\text{CO}}_{3}^{•-}$ ) (Reactions 10–12). Among these species, the nitrogen trioxide radical is the strongest oxidant (2.5 V vs. NHE) (Armstrong et al., 2015). Despite being weaker than the hydroxyl radical, they can oxidize on one-electron transfer reactions a wide variety of organic and inorganic molecules, such as phenols, anilines, sulfur compounds, and some metal ions (Ross and Neta, 1982; Fang et al., 2014; Rayaroth et al., 2022).

$$\text{BDD}\left({\text{OH}}^{*}\right)+{\text{NO}}_{2}^{-}⇄\text{BDD}\left({\text{NO}}_{2}^{•}\right)+{\text{OH}}^{-}$$ (10)

$$\text{BDD}\left({\text{OH}}^{*}\right)+{\text{NO}}_{3}^{-}⇄\text{BDD}\left({\text{NO}}_{3}^{•}\right)+{\text{OH}}^{-}$$ (11)

$$\text{BDD}\left({\text{OH}}^{*}\right)+{\text{CO}}_{3}^{2-}⇄\text{BDD}\left({\text{CO}}_{3}^{•-}\right)+{\text{OH}}^{-}$$ (12)

**FIGURE 6:**
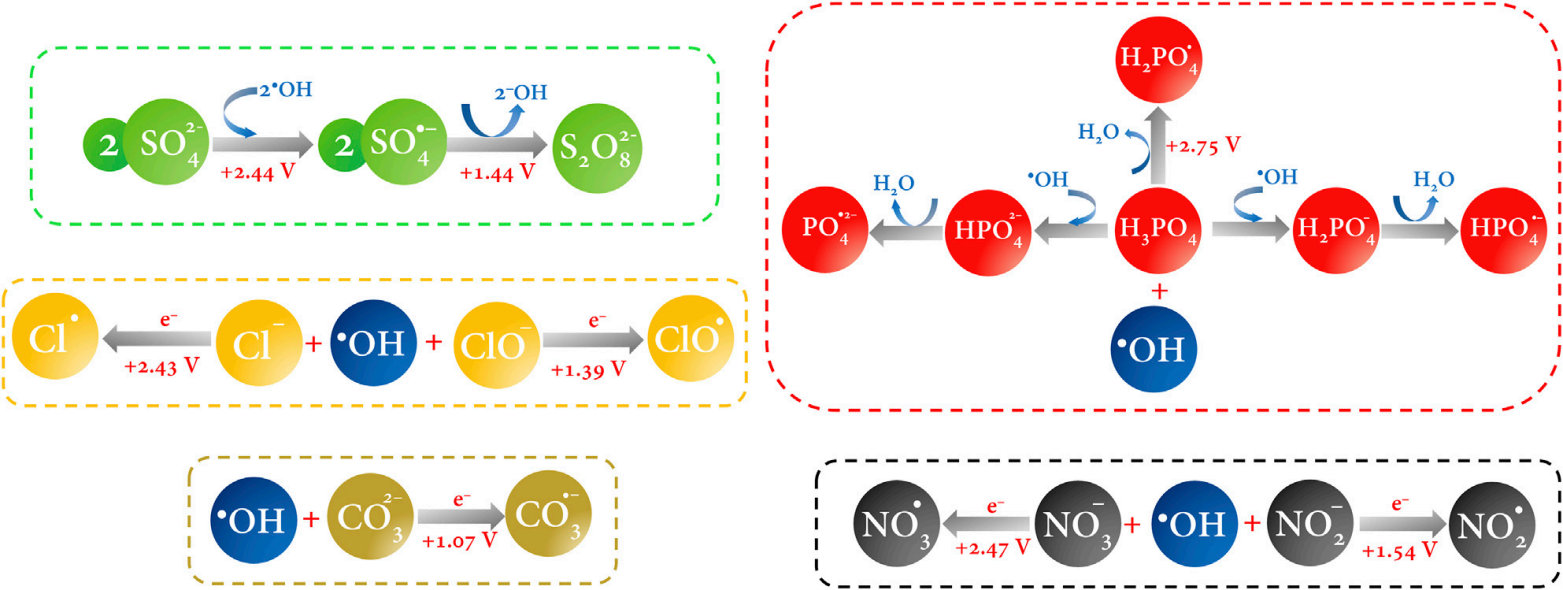
HORS generation pathway by reaction of the ^•^OH in a one-to-one electron transfer. Redox potential values are reported vs. NHE and were taken from the work of Armstrong et al. (2015).

In this section, we left for a while the main aim of this work and tried not to digress in exposing all the species that can exist in an electrochemical reaction medium; instead, we emphasized the main highly reactive oxidizing species of each type depending on the electrolyte (Figure 6). In practice, it is obvious that after the ROS and, especially, ^•^OH, the other species are the consequence of the redox reaction of the latter with the ions in solution; *i.e.,* the other species can be regarded as trapping hydroxyl radicals. This is because ^•^OH is not selective and reacts simultaneously with whoever stabilizes its unpaired electron most quickly and efficiently. All these species highlighted above compete in electrocatalysis, and attributing the major contribution to pollutant degradation to just one of them would be a mistake. Instead, emphasis should be placed on controlling the other variables that take place in electrochemical oxidation.

## 3 Beyond the lab

### 3.1 Limiting current and limiting potential

All the previous analyses can be carried out successfully with accuracies that slightly deviate from the theory, but when the results are transferred to a scale larger than that of the laboratory, it often involves complications that lead to results that deviate greatly from those predicted at the micro-scale, but are not wrong for the most part. These noticeable differences, which are often found at a real scale, are mainly due to the electrochemical cell and the intrinsic energy consumption. That is, when testing electrode behavior, electrolyte effects, and cell configuration in the laboratory, it is common to use simple systems on account of having known and small electrode areas, solution volumes no larger than 25 mL, and simple cell configurations, usually batch type (Bard et al., 2022). In the pre-pilot scale, all the dimensions of the upstream electrochemical components are increased. This increase has important implications for current and potential density. Thus, at scales larger than the laboratory, it is common to have control of the experimental limiting current, *i.e.*, the current that maximizes the charge transfer efficiency of the redox reaction occurring at the electrode surface. Typically, the limiting current can be found in electrooxidation systems as a function of time in the chemical oxygen demand (COD) (Eq. 2) (Wei J. J. et al., 2011; Ochoa-Chavez et al., 2018).

$$i_{\lim}=i_{\lim}\left(t\right)=4Fk_{m}\text{COD}\left(t\right),$$ (2)

where i_lim_ is the limiting current, F is Faraday’s constant, and k_m_ is the mass transfer coefficient. For microelectrode or rotating disk arrays, the limiting current can be calculated by SCV in known redox systems, and its procedure can be found elsewhere (Soares et al., 2020; Bard et al., 2022).

Then, when scaling a system, it is not strange that current densities or overpotentials reach values higher than those predicted by Tafel curves or voltammetric profiles in various electrolytes (Moreira et al., 2017). This is because the diffusion flow of the species within the solution towards the electrode surface changes and, therefore, the energy barrier to be overcome also increases; on the other hand, the potential loss by Ohmic drop also increases significantly due to the resistance of the solution and the micro- and nano-bubbles that evolve on the surface, as well as the presence of the target molecule to be electrochemically oxidized (Petrii et al., 2007; Lee and Bazylak, 2021; Zheng, 2023). In practice and when disk electrodes are not available, the limiting potential and its limiting current are controlled in a non-analytical way (at least for now); *i.e.*, when the electrooxidation test is carried out, the electrode potential is progressively increased, and the current density circulating in the reactor is controlled, and when the proportionality between the potential and current is lost, the limiting current has been exceeded; in other words, the maximum potential value that the system supports so that the charge transfer is also maximum corresponds to the current limit where the process is optimized.

### 3.2 Instantaneous current efficiency and energy consumption

Additionally, another parameter of interest when scaling certain processes is the determination of the instantaneous current efficiency (ICE), which gives us information regarding the optimum range of applicability of a process (Comninellis and Pulgarin, 1991; Xing et al., 2018); this can be calculated by the following equation:

$$\text{ICE}=\frac{\left[{\text{COD}}_{t}-{\text{COD}}_{t+∆t}\right]\text{FV}}{8I∆t},$$ (3)

where (COD)_t_ and (COD)_t+Δt_ are the chemical oxygen demand at times t and t + Δt (in g O_2_ dm^-3^), F is Faraday’s constant, V is the volume of electrolyte (dm^3^), and I is the applied current (A).

Furthermore, the energy consumption of the process is also relevant since it gives us a picture of the efficiency from the perspective of energy expenditure and also helps us to optimize not only the parameters but also the resources (Brillas et al., 2009). Energy consumption is calculated by the following equation:

$$\text{Energy}\;\text{consumption}\;\left(\text{kWh }m^{-3}\right)=\frac{E_{\text{cell}}\text{It}}{V_{s}},$$ (4)

where E_cell_ is the cell voltage (V), t is the electrolysis time (h), and I is the current passing through the cell (A). Therefore, the cost of the process can be obtained according to the following equation:

Cost of process=Energy consumption×Cost per kWh. (5)

**SCHEME 1:**
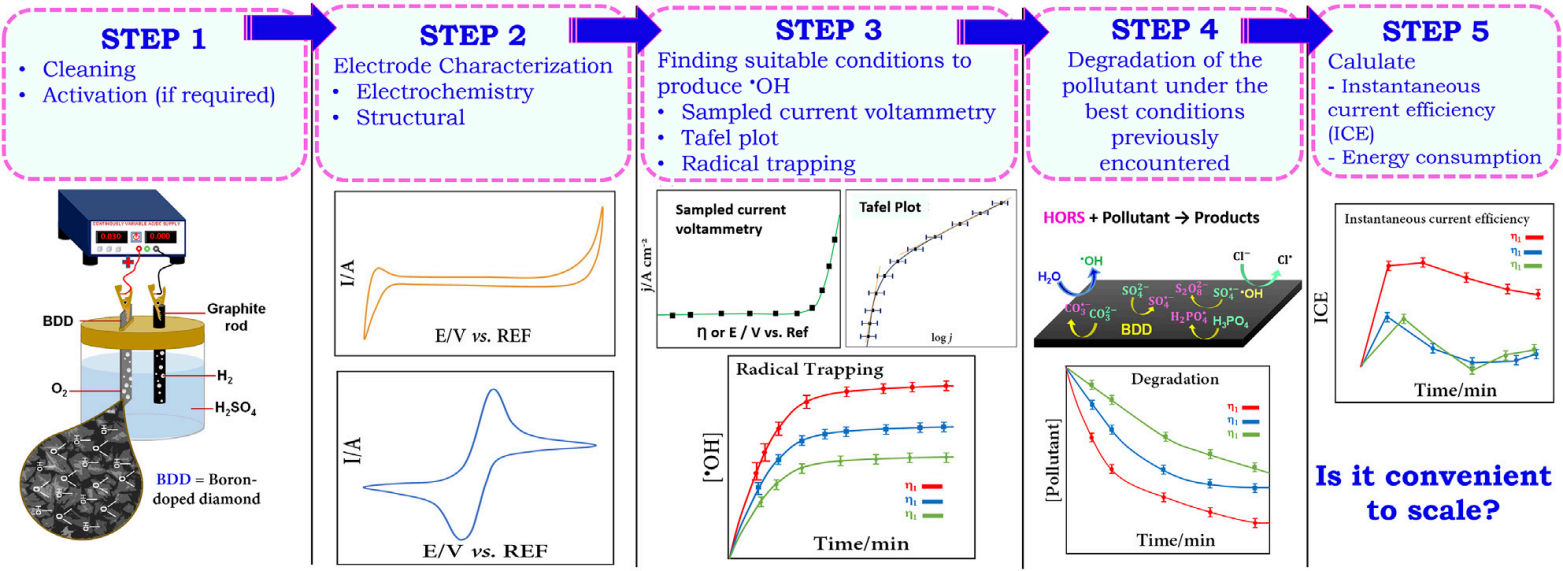
Systematic step-by-step approach for the investigation of HORS on BDD.

In summary, Scheme 1 presents a systematic representation of the steps that we aimed to introduce so far and serves as a guide for the user; we emphasize that this is a preliminary approach to get to know our working electrode in the presence of a suitable electrolyte. We encourage the authors to include new electrochemical and non-electrochemical techniques to gain a deeper understanding of the electrode materials to be investigated.

The following is a specific case study involving BDD in the search for the optimal potential range for the generation of HORS, applying the reasoning that has been shown so far in the search for the prime parameters by carrying out electrochemical tests that can be handled without major problems in any electrochemical laboratory.

## 4 Case study: BDD in a Na_2_SO_4_ medium

Quite recently, Lu et al. (2023) highlighted the most recent advances in the effects of electrolytes in a solution for electrochemical processes and discussed their effects on different reactions of interest in electrochemistry, such as oxygen and hydrogen evolution reactions (HER and OER, respectively) and oxygen and CO_2_ reduction reactions (ORR and CO_2_RR, respectively). Nevertheless, to date, no review has been found in the literature that addresses, in a simplistic fashion and with an experimental approach, the investigation of ionic species in a solution and their participation in electrochemical oxidation as a guide for beginners. In this section, a real experimental example is presented as a case study that evaluates the behavior of BDD in Na_2_SO_4_ and other electrolytes of interest, emphasizing the steps that should be followed if one wishes to begin in this field.

Anhydrous sodium sulfate (N_2_SO_4_ 99%) was purchased from J.T. Baker, *N*,*N*-Dimethyl-4-nitroso-aniline (for synthesis) was purchased from Merck, and amoxicillin was purchased from Sigma-Aldrich. All chemicals were used without further purification. BDD electrodes were purchased from CONDIAS and were cut into 25 mm × 15 mm rectangular plates, and an effective bipolar electrode area of 6.0 cm^2^ in a cell volume of 60.0 mL was used for all experiments. The BDD was previously electrochemically activated by anodic polarization for 10 min in 0.5 mol L^-1^ sulfuric acid (H_2_SO_4_ 95%–97%, Merck) at 0.1 Acm^-2^ in a BK Precision AC/DC power supply. All electrochemical assays were carried out on a BioLogic potentiostat/galvanostat workstation in a typical three-electrode cell. The BDD served as the working electrode, Ag/AgCl served as the reference, and a graphite rod served as the counter electrode. For the degradation of amoxicillin (40 μM), a two-electrode system was used, with a BDD as the anode and a Pt mesh as the cathode.

### 4.1 Cyclic voltammetry and polarization curves

**FIGURE 7:**
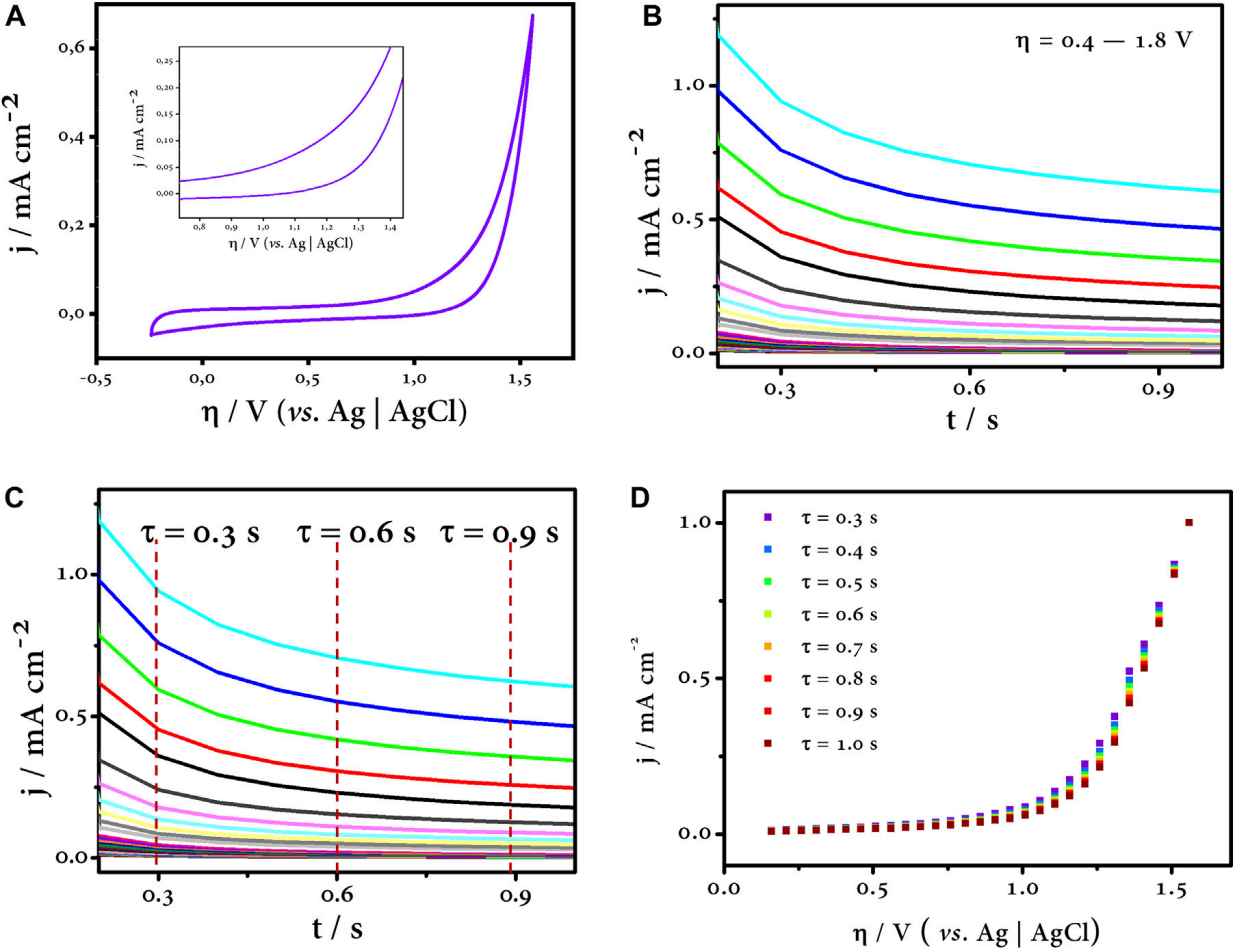
CV profile **(A)** and polarization curves **(B)** at oxidation overpotentials. The time constants were selected **(C)** for plotting the η vs. j curve by sampled current voltammetry **(D)**. All assays were carried out on a typical three-electrode system (WE: BDD, CE: graphite rod, and RE: Ag/AgCl in Na_2_SO_4_ 0.1 mol L^-1^).

Once the electrode or electrode material to be evaluated—BDD in this case—has been selected, the electrolyte medium in which the behavior of the electrode is to be investigated is chosen without any additional species in the solution. Therefore, the first step is to perform cyclic voltammetry at oxidative potentials—actually overpotentials—in the chosen medium; our interest is in Na_2_SO_4_ for reasons already discussed earlier. The CV gives us a first insight into the potential where oxygen evolution is kinetically and thermodynamically predominant. The inflection point of the oxidation branch of the CV indicates the potential where the transition from one electron transfer process to another is likely to occur and is likely to be the potential where most intermediate reactions compete, which is approximately 1.20 V vs. Ag/AgCl according to Figure 7A. Then, by chronoamperometry, we determine the polarization curves at the oxidation potentials determined in the previous CV profile (Figure 7B).

### 4.2 Sampled current voltammetry and η vs. j curves

After completing the polarization curves at the oxidation overpotentials, the next step is to choose a time interval, called time constant (τ), in which the process is controlled by charge transfer (Bard et al., 2022). For this purpose, the SCV is a useful tool that allows us to control this phenomenon when a rotating disk electrode is not available (Soares et al., 2020). Typically, it consists of choosing current values at small time constants and constructing η vs. j curves (Figures 7B, C). In this range, the process is kinetic-controlled, and the influence of diffusion, *i.e.*, mass transfer, is limited, so the effect of the electrode capacitance does not affect our test, and all measurements are focused entirely on the Faradaic current (Soares et al., 2020; Rodríguez and Denuault, 2021; Bard et al., 2022). Finally, Figure 7D shows all the η vs. j curves corresponding to different time constants, and all of them fit the same trend.

### 4.3 Tafel plot

**FIGURE 8:**
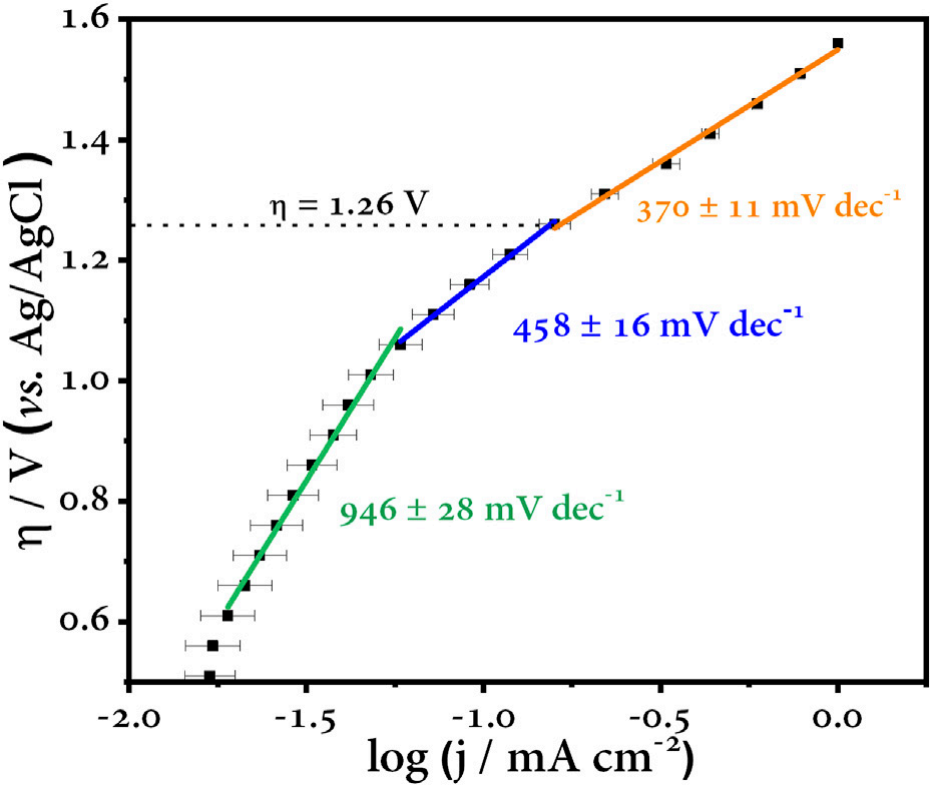
Tafel plot and slopes corresponding to the oxygen evolution reaction.

As discussed in previous sections, the Tafel analysis is useful to evaluate the kinetics of an electrochemical process. In this sense, and summarized in the last step of our survey, the Tafel curve is constructed by plotting the logarithmic function of the current *versus* the overpotential (Figure 8). Three zones can be distinguished in this curve (highlighted by the straight lines). Each region of the curve has an associated slope, and this is the Tafel slope. The green line corresponding to the first zone has a slope value of 946 mV dec^−1^, and this region is purely capacitive (it can be checked with the CV profile). The overpotential values up to about 1.1 V vs. Ag/AgCl are potentials where the double layer of the electrode—at the solid–liquid interface—is charged, and all this charge is stored in this imaginary region of the electrode, the inner Helmholtz plane (Bard et al., 2022). The next region illustrated by the blue line has a Tafel slope of 458 mV dec^−1^, and this sharp change in slope indicates that another process(es) is taking place at the electrode surface. Finally, the last zone highlighted by the orange line has a slope of 370 mV dec^−1^. This region plotted at overpotentials above 1.3 V vs. Ag/AgCl refers to the last process. This zone is distinctive as oxygen evolution reaction is predominant (Kapałka et al., 2008). This is self-evident as the error bars in this last region are almost undistinguishable, *i.e*., the current and potential values do not oscillate to a large extent as long as the test is run again. The opposite is true for the previous zones. The intermediate zone is of interest to us as the former is easily discarded because of its capacitive contribution. The intermediate zone allows us to have an overview of the processes that can take place at these potential values. Since we know the chemical nature of the medium in which the test was carried out, we can say that this region is dominated by the generation of HORS that compete with the ^•^OH, our main oxidant.

However, despite our efforts, we cannot be certain of the exact potential at which the most highly oxidizing species can be harnessed for any given process, and to do so would be a tremendous mistake. Instead, these steps allow us to determine a wide range of potentials and discriminate those that do not contribute to our process to save us errors, time, and costs of unnecessary laboratory testing. Quantitative assays such as the quantification of reactive species or degradation of a known contaminant should be added to this procedure to extend this assumption and have a more adequate approach to our purpose; with this, we will have approached our search for the optimal experimental conditions to achieve adequate efficiencies in our advanced electrochemical oxidation process. To experimentally verify the overpotential region where the highest amount of ^•^OH is generated, a radical trapping study was performed, as shown below.

### 4.4 Trapping of radicals

**FIGURE 9:**
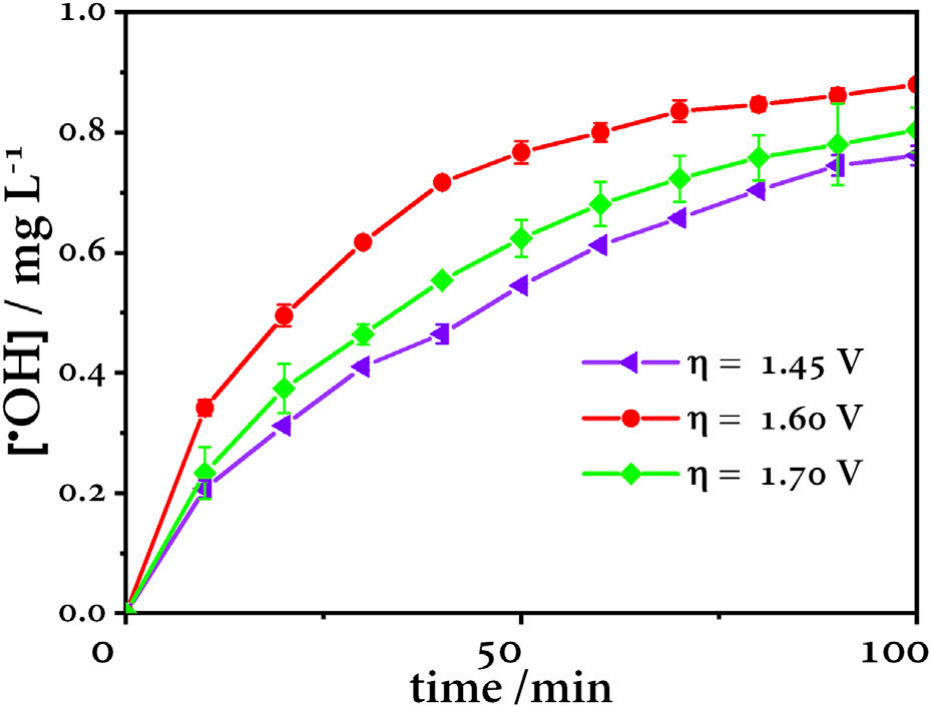
Effect of anode overpotential in the production of ^•^OH using a BDD anode, Pt cathode, and Ag/AgCl reference electrode.

The ^•^OH concentration was quantified by the N,N-Dimethyl-4-nitroso-aniline (RNO) method (Ochoa-Chavez et al., 2018). Anode overpotentials were chosen based on the Tafel plot at values above 1.26 V vs. Ag/AgCl, according to Figure 8. At an anodic overpotential value of 1.60 V vs. Ag/AgCl, the highest concentration of ^•^OH is generated, while at 1.45 V vs. Ag/AgCl, the concentration is lower because the applied potential is not enough to generate the maximum concentration of ^•^OH. On the other hand, at 1.70 V, the concentration decreases because OER is the predominant reaction (Figure 9).

### 4.5 Amoxicillin degradation

**FIGURE 10:**
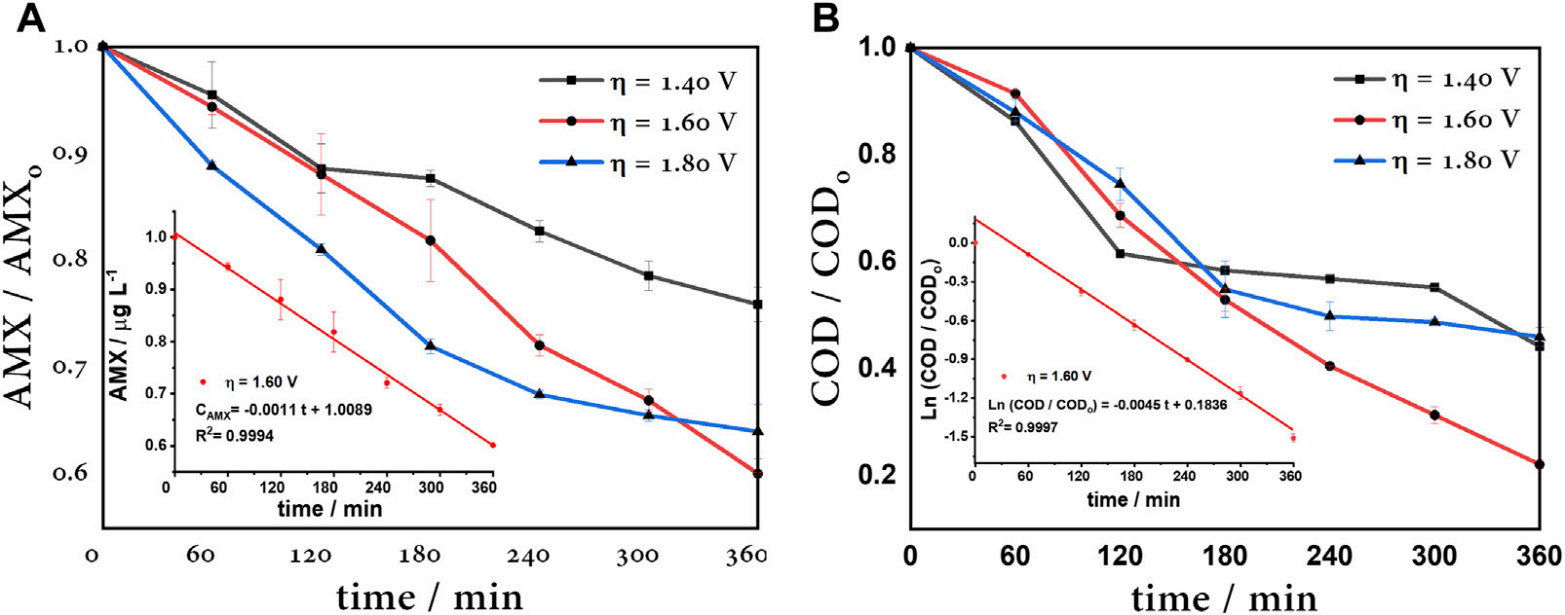
**(A)** 40 μM AMX degradation at different overpotentials. Inset: zero-order kinetic of amoxicillin removal. **(B)** COD of 40 μM AMX degradation at different overpotentials. Inset: pseudo-first-order kinetic of COD removal, using a BDD anode, Pt cathode, and Ag/AgCl reference electrode.

vIn order to verify that the higher ^•^OH radical production matches a maximum degradation efficiency, the electrooxidation of amoxicillin (AMX 40 μM) was tested. The electrochemical degradation of amoxicillin was evaluated under the best ^•^OH production conditions, as discussed above. The degradation was followed by UV–Vis spectroscopy and chemical oxygen demand (COD). According to Figure 10A, at η = 1.40 V, the competition between the ^•^OH electrogeneration (E° = 2.43 V vs. SHE) and formation of sulfate species ${\text{SO}}_{4}^{\;•\;–}$ (E° = 2.44 V vs. SHE) and ${S_{2}O}_{8}^{2-\;}$ (E° = 1.44 V vs. SHE) is expected to be carried out (Armstrong et al., 2015); this agrees with the first minutes of electrolysis where amoxicillin degradation decreases rapidly and then remains unchanged. This is because sulfate species are not able to completely mineralize highly stable small organic molecules such as formic acid and oxalic acid (k = 1.3×10^8^ and 1.4×10^6^ L mol^-1^ s^-1^, respectively), reaching 24.1% AMX concentration removal and 55.8% COD removal in 360 min of electrolysis (Figure 10B). On the other hand, at η = 1.60 V, the highest amoxicillin removal is achieved; this potential corresponds to the highest mineralization, and these removals decrease linearly until reaching 39.9% and 77.9%, respectively; therefore, this overpotential obtains the highest ^•^OH production. Whereas, when overpotential increases, at η = 1.80 V, the removal behavior is similar as that at 1.40 V, and in this case, the OER is predominant, obtaining only 36% amoxicillin removal and 54% COD removal.

In summary, Table 1 shows the kinetic parameters, where rate constants for amoxicillin degradation were adapted to a zero-order kinetic model and a pseudo-first-order kinetic model to COD removal. The COD constant rate of 1.60 V was almost two times greater than others’ overpotentials; therefore, this is considered the optimal anode overpotential. In this case, this is the overpotential corresponding to the limit current, *i.e.,* the current associated to the maximum capacity to degrade AMX.

**TABLE 1:** Kinetic parameters of amoxicillin degradation.

η/V vs. Ag/AgCl	AMX remotion (%)	k_amx_/min × 10^–3^	COD remotion (%)	k_COD_/min^-1^ × 10^−3^
1.4	24.1	0.7	55.8	2.0
1.6	39.9	1.1	77.9	4.5
1.8	36.0	1.0	54.0	2.3

AMX, amoxicillin; η, overpotential; COD, chemical oxygen demand; k_AMX_, zero-order kinetic constant; k_COD_, pseudo-first-order kinetic constant.

### 4.6 Instantaneous current efficiency and energy consumption

**FIGURE 11:**
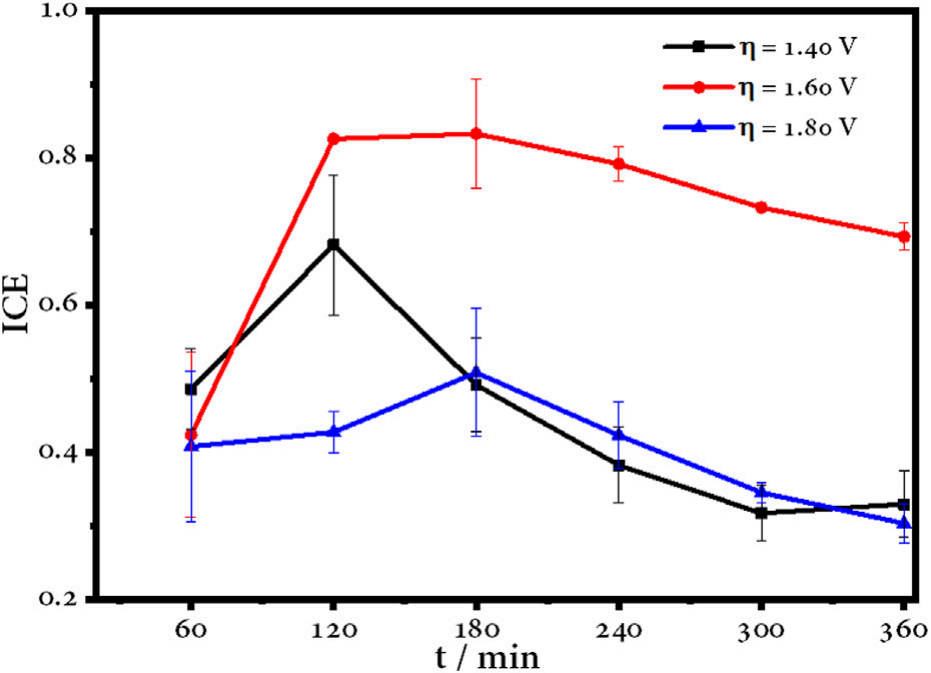
Instantaneous current efficiency over time for the electrochemical degradation of amoxicillin using a BDD electrode.

Figure 11 shows the evolution of the instantaneous current efficiency (ICE) with respect to electrolysis time, where maximum values of ICE are reached in the first hours, after which it decays due to the oxidation process and the formation of organic compounds. In the case of 1.60 V, it can be observed that after 2 h, the ICE remains stable, so the generation of ^•^OH is continuous and allows progressive degradation, as it was discussed previously. Nevertheless, the ICE of the other overpotentials decreases drastically.

On the other hand, Table 2 shows the energy consumption and operating cost for the degradation of amoxicillin at different overpotentials. The cost of operation was calculated based on the cost per kilowatt-hour in Ecuador, which is 0.096 USD until March 2023 (Global Petrol Prices, 2023).

**TABLE 2:** Parameters of efficiency for the amoxicillin electrooxidation during a 6 h reaction.

η/V vs. Ag/AgCl	ICE	Energy consumption/kWh m^−3^	Cost/USD m^−3^
1.40	0.33	0.252	0.024
1.60	0.69	0.224	0.022
1.80	0.30	0.324	0.031

η, overpotential; ICE, instantaneous current efficiency.

The results showed that the energy used during the electrolysis under the optimal overpotential was 0.224 kWh m^-3^, and the operation cost was 0.022 USD m^-3^, which represents the lowest cost among the other potentials tested and is in mutual agreement with the most efficient potential of the degradation process.

## 5 Conclusion and outlook

Properly understanding the behavior of an electrode in a specific system can sometimes be a complicated endeavor, even more so when one does not know the charge transfer processes that may or may not take place in the medium under study and when this medium is a complicated mixture of organic and inorganic species. Electrochemical oxidation is a useful and efficient technology for many remediation applications if applied correctly, *i.e*., having adequate knowledge and control over the parameters that influence the process, both internal and external. In this sense, this work attempted to provide a step-by-step guide, particularly for beginners, on the tests to be considered in the investigation of electrodes and new electrode materials for advanced electrochemical oxidation processes, with emphasis on the range of anodic potentials where the generation of highly reactive oxidizing species predominates, which are priceless in these processes. It is not our intention that this guide should be followed rigorously, but rather that it should serve as a support for those who wish to get started in this field, adjusting these steps to their research objectives. The study of the kinetic behavior of BDD in different electrolytes by SCV, the Tafel plot, and trapping of radicals is essential to achieve results of this magnitude. A detailed study of the effect of the electrolyte is in progress in our research group.

Although this guide is particularly focused on the BDD, it is not limited to this electrode. This guide can be followed to investigate a new electrode material from an electrochemical point of view at first glance. In addition to the steps proposed here, the reader is free to implement other electrochemical techniques that suit his or her research. For example, electrochemical impedance spectroscopy is important when specific reaction mechanisms need to be studied and can provide valuable information about a particular electrode; however, this technique is beyond the scope of this work.

## Data Availability

The original contributions presented in the study are included in the article/Supplementary Material; further inquiries can be directed to the corresponding author.
